# Hepatoprotective Effects of Crude Stem Bark Extracts and Solvent Fractions of *Cordia africana* against Acetaminophen-Induced Liver Injury in Rats

**DOI:** 10.1155/2022/1449286

**Published:** 2022-03-29

**Authors:** Gudeta Duga Geresu, Shemsu Umer, Mahlet Arayaselassie, Genet Ashebir, Eyasu Makonnen

**Affiliations:** ^1^Department of Pharmacy, College of Medicine and Health Sciences, Ambo University, Ambo, Oromia, Ethiopia; ^2^Department of Pharmacology and Clinical Pharmacy, College of Health Sciences, Addis Ababa University, Addis Ababa, Oromia, Ethiopia; ^3^Department of Pathology, College of Health Sciences, Addis Ababa University, Addis Ababa, Oromia, Ethiopia; ^4^Ethiopian Public Health Institute, Addis Ababa, Oromia, Ethiopia; ^5^Center for Innovative Drug Development and Therapeutic Trials for Africa (CDT-Africa), Addis Ababa University, Addis Ababa, Oromia, Ethiopia

## Abstract

*Cordia africana* Lam (Boraginaceae) is widely used in Ethiopian folk medicine for the treatment of different types of liver disorders. Thus, this study aimed to investigate the hepatoprotective effects of an aqueous (CAAE), 80% methanol extracts of *C. africana* stem bark (CAME), and the solvent fractions of the methanol extract against acetaminophen (APAP)-induced liver injury in rats. Acute toxicity test and APAP-induced lethality test were done on mice of either sex, while APAP dose selection test was done on female rats. Male rats were used for hepatoprotective experiments and the liver injury was induced using 2 g/kg APAP given orally. Serum levels of the liver enzymes and total bilirubin (TB), as well as lipid profiles, were determined. Histopathological examination of the liver tissues was also conducted to confirm the findings of biochemical analysis. Intraperitoneal (i.p.) sodium pentobarbital (SPB)-induced sleeping duration was also used to determine the protective effect of the test substances. Oral administration of APAP resulted in a significant increase in serum levels of alanine aminotransferase (ALT), aspartate aminotransferase (AST), alkaline phosphatase (ALP), TB, low-density lipoprotein (LDL), total cholesterol (TC), and triglycerides (TGs) and decrease in serum high-density lipoprotein (HDL). Administration of the standard drug, silymarin 100 mg/kg, extracts at doses of 100, 200, and 400 mg/kg and fractions at the dose of 400 mg/kg reversed the serum levels of all parameters to normal. CAME exerted a significant dose-dependent hepatoprotective effect in terms of ALT and AST, while CAAE significant dose-dependent hepatoprotective effect was in terms of AST, ALP, and TGs. The protective effect of the extracts and fractions was also confirmed by histopathological investigations and SPB-induced sleeping time. From the results of the present study, it can be concluded that *C. africana* stem bark aqueous, 80% methanol crude extracts, and solvent fractions of the methanol extract showed hepatoprotective effects.

## 1. Introduction

The liver is the largest internal organ with an extraordinary spectrum of functions. Multidimensional functions and its strategic location make it prone to many diseases. Liver diseases are largely neglected health issues in developing countries that carry the highest burden [1, 2]. Many drugs, toxins, and herbal medicines have been reported to cause liver injury and drugs account for 20–40% of all instances of hepatic failure. However, until today, there is no precise and effective therapeutic drug for the management of diseases. Moreover, the currently used drugs for the management of liver diseases are costly and have many unwanted effects. Thus, there is a need to search for new drugs that are safe, effective, and affordable.

Due to cultural acceptability, relatively low cost of traditional medicine, and limited access to modern health care facilities, about 80% of the population in Ethiopia relies on TM for their primary health care [3]. Cordia is a pantropical genus of flowering plants that belong to the family of Boraginaceae. Many species of the genus Cordia such as *Cordia dichotoma*, *Cordia latifolia*, *Cordia macleodii*, *Cordia myxa*, *Cordia rothii,* and *Cordia obliqua* are used in Ayurveda, Unani, and Siddha systems of medicine. The major reported pharmacological activities of the extracts and isolated compounds include anti-inflammatory, antioxidant, larvicidal, hepatoprotective, analgesic, antimicrobial, and antidiabetic [4]. *C. dichotoma*, *C. myxa*, *C. obliqua*, *Cordia sebestena,* and *C. macleodi* are among species of the genus Cordia that confirmed the hepatoprotective effect in animal studies [5–10].

In Ethiopia, *Cordia africana* is known by different vernacular names such as “Wanza” in Amharic, “Waddeessa” in Afan Oromo, and “Ahwi” in Tigrigna with different medicinal uses [11]. Many ethnobotanical reports [12–16] have shown that different parts of the plant are traditionally used for the liver diseases. Secondary metabolites found in the stem bark of *C. africana* such as phenols [17], flavonoids [18], alkaloids [19], saponins [20], and tannins have shown hepatoprotective activity in other studies. Moreover, the plant stem bark extract has shown *in vitro* antioxidant [21] and *in vivo* anti-inflammatory [22] activities, both of which play a great role in preventing the liver diseases caused by oxidizing agents and consequent inflammatory reactions. Due to its prominent antioxidant activity, this plant has been considered as one of the African plants with potential hepatoprotective activity [10]. Consistent with ethnomedicinal reports, hepatoprotective activities shown by other plants of the same genus and experimentally confirmed antioxidant and anti-inflammatory activities of the plant itself made *C. africana* to be screened for hepatoprotective activity in the animal models. Therefore, this *in vivo* study used APAP-induced liver injury in the animal model to investigate hepatoprotective effects of *C. africana* stem bark extracts and solvent fractions.

## 2. Materials and Methods

### 2.1. Materials

Methanol (99.8% for HPLC-Gold-Ultra Gradient, CARLO ERBA Reagent), chloroform (99.8% for HPLC-Gold-Ultra Gradient, CARLO ERBA Reagent), n-butanol (99%, LOBA CHEMIE Pvt. Ltd.), Tween-80, distilled water, silymarin tablets (Liverubin, Alchem International Pvt. Ltd.), APAP tablets (Para-Denk 500, Denk Pharma), normal saline (0.9% sodium chloride solution), 10% buffered neutral formalin solution (formaldehyde 35%, Sigma-Aldrich, Co.), paraffin, xylene, hematoxylin solution, 2% eosin solution, and closed reagent cassettes (Roche Diagnostic, Indianapolis) for all ALT, AST, ALP TB, HDL, LDL, TC, and TGs, bought from Germany were used.

### 2.2. Plant Materials

The stem bark of *C. africana* was collected from Ambo, West Shoa Zone, Oromia, about 114 kilometers West of the capital city, Addis Ababa, Ethiopia in December, 2019. The plant was identified and authenticated by Dr. Getachaw Adis, a botanical taxonomist at the Ethiopian Public Health Institute, Addis Ababa, Ethiopia. The specimen with voucher number (GD-001) was kept in the herbarium of the Ethiopian Public Health Institute Traditional and Modern Medicine Research Directorate for future reference.

### 2.3. Preparation of an Aqueous and 80% Methanol Extracts

The stem bark of *C. africana* was dried under the shed for two weeks after washing with tap water to remove any dirty materials, chopped, and grinded by a mechanical grinder into a coarse powder. Methanol extract of *C. africana* (CAME) was prepared by macerating 600 grams of the powder in 80% methanol (99.8% for HPLC-Gold-Ultra Gradient, CARLO ERBA Reagent) in the ratio of 1:7 (one gram of the powder in 7 ml of 80% methanol) for 72 hrs with occasional shaking and the extracts were combined. To prepare an aqueous extract of *C. africana* (CAAE), an aqueous decoction, 500 grams of the powder was weighed, boiled with distilled water for 30 minutes, and allowed to be cooled. The extraction methods were used according to traditional use and the extracts were separately filtered with gauze and then filter paper using a filter-aided vacuum. The filtrate of methanol extract was concentrated by a rotary evaporator under reduced pressure at a temperature not exceeding 40°C in a heating bath to obtain a 7.90% yield. An aqueous filtrate was kept in a refrigerator to freeze the water, which was removed by a lyophilizer, and a 7.00% yield was obtained.

### 2.4. Preparation of Solvent Fractions

Since CAME exerted a better hepatoprotective effect at the medium dose, it was subjected to solvent-solvent fractionation using solvents of different polarities such as chloroform, n-butanol, and water using a separator funnel. The organic solvents were removed under reduced pressure using a Rotavapor. An aqueous fraction was freeze dried following the same procedure used for the aqueous crude extract using a lyophilizer. The resulting semisolid masses of fractions were placed in a vacuum oven at 30°C for about 48 h to remove any residual solvent and stored in a desiccator under the same conditions as that of the crude extracts until use.

### 2.5. Experimental Animals

Rats and mice of either sex weighing 150–215 g (8–10 weeks old) and 30–35 g (6–7 weeks old), respectively, were obtained from Addis Ababa University's pharmacology laboratory. All experimental procedures were conducted in accordance with the standards set forth in the guidelines for the care and use of experimental animals by the Committee for Purpose and Control of Supervision of Experiments on the Animals [23]. The animals were acclimatized to the animal house conditions for a period of seven days before experimentation to minimize the effects of environmental changes and stress associated with transportation-induced physiological changes such as cardiovascular, immunological, and neural and endocrine changes. Then, they were randomized into various groups and housed in groups of five to ten animals in polypropylene cages, lined with softwood flakes as bedding at room temperature. They were allowed free access to standard pellets, except during the fasting period, and tap water regularly.

The animals were given code marks using a permanent marker to separate them as per their body weight. The standard drugs, plant extracts, fractions, and vehicles were administered using oral gavages of appropriate size. All administrations were recorded on the prepared checklist to prevent missing or double administration. During the procedures, the animals were anaesthetized by chloroform intranasally to prevent distress and finally, the anaesthetized animals were sacrificed through cervical dislocation.

### 2.6. Acute Oral Toxicity Test

Acute toxicity test on the stem bark extracts of *C. africana* was carried out based on the limit test recommendations of Organization for Economic Co-Operation and Development Guideline-425 [24], on mice of either sex. Two groups of the animals containing five mice each were used, that is, group I for CAME and group II for CAAE. Mice were fasted for four hours prior to the experiment and allowed free access to water *ad libitum* during fasting. A single dose of 2000 mg/kg of CAME or CAAE dissolved in 2% Tween-80 in distilled water was administered to each animal using oral gavages. After administration, mice were fasted for two hours and given free access to food and water. The animals were then observed for physical and behavioral changes within 12 h, with special attention during the first 4 h. The major signs of toxicity observed were difficulty in breathing, loss of appetite, general weakness, irritability, writhing, loss of motor coordination, muscle relaxation, sedation, and deep sleep. They were further observed every day for 14 days to see any mortality and the test was repeated with 3000 mg/kg of extract.

### 2.7. Acetaminophen-Induced Lethality Test

APAP lethality test was conducted according to the method described by Gilani et al. [25]. Briefly, the animals were grouped into three with 10 per group and fasted for twelve hrs with free access to water prior to extract administration and for one hour after administration of APAP. Group I served as a control and received the vehicle, 2% of Tween-80 dissolved in distilled water; groups II and III received 400 mg/kg of CAME and CAAE, respectively, dissolved in 2% Tween-80. One hour later, all the animals received the lethal dose of APAP (1 g/kg). The number of deaths within 24 hours was recorded and percent protection was calculated using the following formula:

(1) $$\begin{array}{c} \text{\% Protection}=\frac{a}{b}\times 100, \end{array}$$

where *a* is the number of surviving animals 24 hours after administration of a lethal dose of APAP and *b* is the number of the animals in the group at the beginning.

### 2.8. Acetaminophen Dose Selection Test

The test was aimed to determine the optimum dose of APAP used for induction of the liver injury. Three different doses of APAP, 640 mg/kg [26], 1 g/kg [27], and 2 g/kg [28], were taken from three different scientific papers. Four groups of the animals containing five female rats each were used for the test. Group I served as a control and received normal saline (NS), 10 ml/kg. Groups II–IV received 640 mg/kg, 1 g/kg, and 2 g/kg of APAP, respectively. All the animals were anaesthetized with chloroform 48 h after APAP administration, and 3–4 ml blood samples were taken through cardiac puncture. Blood samples were put into serum separator tubes containing a clot activator and allowed to clot at room temperature for 1 hr. Then, it was centrifuged at 3000 revolutions per minute for 15 minutes at 4°C, and serum was taken to determine the levels of alanine aminotransferase (ALT) and aspartate aminotransferase (AST). The dose inducing significant biochemical changes without causing any physical damage was selected to be used for the main test. Accordingly, experimental liver damage was induced by administering a single dose (2 g/kg) of APAP [29].

### 2.9. Hepatoprotective Activity against Acetaminophen-Induced Liver Damage

Nine (I–IX) groups containing five animals each were used for the hepatoprotective test of the crude extracts (an aqueous and 80% methanol extracts). This included the normal control (received 2% Tween-80), standard drug group (received silymarin 100 mg/kg in 2% Tween-80), CAME groups (received 100, 200, or 400 mg/kg), CAAE test groups (received 100, 200, or 400 mg/kg), and negative control group (received 2% Tween-80). Dosing was according to the one used for acute toxicity test, that is, 2000 mg and previous studies [30]. All test substances and standard drug were freshly prepared by dissolving in 2% Tween-80 and given daily for ten days. On day ten, all the animals in groups II–IX were given 2 g/kg APAP in NS one hour after administration of the last dose, while group I animals received the vehicle, NS.

After identifying the extract with better activity, fractionation of CAME was done using different solvents. Thus, the fractions were also investigated for their hepatoprotective activity using a similar procedure mentioned above. Accordingly, there were three groups, containing five animals each, which received 400 mg/kg aqueous fraction of methanol extract of *C. africana* (CAAF), 400 mg/kg butanol fraction of methanol extract of *C. africana* (CABF), and 400 mg/kg chloroform fraction of methanol extract of *C. africana* (CACF), respectively, for ten days. On the tenth day, all groups received 2 g/kg APAP.

### 2.10. Biochemical Analysis

The levels of the liver enzymes, ALT, AST, alkaline phosphatase (ALP), gamma-glutamyl transferase (GGT), and the level of total bilirubin (TB) were determined. Lipid profiles including total cholesterol (TC), triglycerides (TGs), high-density lipoproteins (HDL), and low-density lipoproteins (LDL) were also evaluated. All biomarkers were analyzed using a fully automated machine, Cobas^®^ 6000. The hepatoprotective activity expressed as percent protection of the extracts and fractions in terms of biochemical parameters was calculated using the following formula:

(2) $$\begin{array}{c} \text{\% Protection}=\left[\left(a-b\right)÷\left(a-c\right)\right]\times 100, \end{array}$$

where *a* is the mean value of marker produced by hepatotoxin (APAP), *b* is the mean value of the marker produced by toxin plus test substances, and *c* is the mean value of the biomarkers produced by the vehicle [31]. *P* values <0.05 were considered significant. Each biomarker was tested according to Maqsood et al. [32].

### 2.11. Histopathological Analysis

Three gross sections of the liver samples were taken from the left lateral lobe, right medial lobe, and caudate lobe, kept in a tissue cassette with the respective identity, and fixed in 10% buffered neutral formalin solution until processing. Sections of 4 *μ*m thickness were taken from each tissue block, stained with hematoxylin for 10 minutes, and counterstained in 2% eosin solution for 20 seconds. The stained slides were coverslipped using DPX mounting medium. Histomorphologic abnormalities were scored based on the type and severity of morphologic changes according to Yahya et al. [33]. Selected images of the sections were captured under a magnification of 10× by Leica DM750 microscope using ICC50 HD camera.

### 2.12. Sodium Pentobarbital-Induced Sleeping Time

The test was done according to the method described by Gilani and Janbaz [34] with some modification on ten groups of male rats containing six per group. Groups I–III served as normal, negative, and positive controls, respectively. Groups I and II received the vehicle, 2% Tween-80, and group III received the standard drug, silymarin 100 mg/kg. Groups IV and V received 400 mg/kg CAME and 400 mg/kg CAAE, respectively. Groups VI to X received CAME, CAAE, CAAF, CABF, and CACF at a dose of 400 mg/kg, respectively. All the animals received four doses of their corresponding vehicle or test substances at twelve-hour intervals. The animals in all groups, except groups I, IV, and V, received 2 g/kg APAP one hour after the last dose of test substances or vehicle. Twenty-four hours after APAP administration, the animals in all groups were given 150 mg/kg of SPB i.p. Data recording formats were prepared for all the animals and each animal was recorded individually. Recorded data include the time of injection, sleep onset (loss of writhing reflex), time of awakening (gain of reflex), and duration of sleep (time between loss and gain of reflex) in minutes.

### 2.13. Statistical Analysis

Data were expressed as mean ± standard error of the mean (SEM). IBM SPSS (Statistical Package for Social Sciences) Statistics for Windows, version 20 software (IBM Corp., Armonk, NY, USA) was used for statistical analysis. Differences between means were determined using analysis of variance (ANOVA) and Tukey post-hoc test with multiple comparisons was used to determine significant differences. *P* < 0.05 was considered statistically significant.

## 3. Results

### 3.1. Acute Toxicity Test

No mortality was observed following administration of both an aqueous and 80% methanol extracts of *C. africana* Lam. (Boraginaceae) stem bark at doses of 2000 and 3000 mg/kg within 14 days. Moreover, no signs of toxicity such as difficulty in breathing, loss of appetite, general weakness, irritability, writhing, loss of motor coordination, muscle relaxation, sedation, and deep sleep were observed during the observation period. Thus, the oral median lethal doses of the extracts are assumed to be greater than 3000 mg/kg.

### 3.2. APAP-Induced Lethality Test

**Figure 1:**
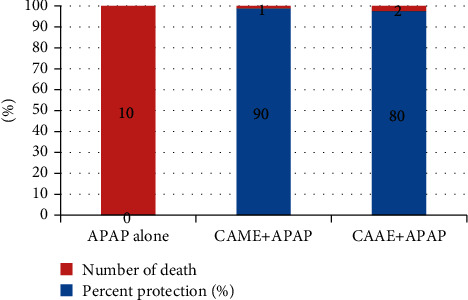
Effects of 80% methanol and aqueous extracts of *C. africana* stem bark on APAP-induced mortality. (APAP-acetaminophen, CAAE-aqueous extract of *C. africana*, and CAME-80% methanol extract of *C. africana*).

Among the animals that received 400 mg/kg CAME or CAAE, only one and two animals died, respectively, within 24 hours of the APAP (1 g/kg) administration, showing the percent protection to be 90% and 80%, respectively (Figure 1). Moreover, these observations were maintained for a one-week duration after which no more observations were made.

### 3.3. APAP Dose Selection Test

The results of APAP dose selection study are presented in Table 1. As can be seen from Table 1, the mean levels of the liver enzymes (ALT and AST) were not significantly increased in the animals that received 640 mg/kg of the APAP compared to controls. About 2000 mg/kg of APAP caused a significant (*P* < 0.05) increase in the serum levels of both ALT and AST and hence, this dose was chosen for the induction of the liver injury.

### 3.4. Biochemical Analysis

#### 3.4.1. Effects of Crude Extracts and Solvent Fractions on the Liver Enzymes and Total Bilirubin

The results of the effects of crude extracts (CAAE and CAME) and solvent fractions of *C. africana* stem bark on the liver enzymes ALT, AST, ALP, and TB in APAP-induced liver damage are presented in Table 2.

As shown in Table 2, the administration of 2 g/kg APAP caused a significant (*P* < 0.05) increases in the serum levels of all these biomarkers compared to the control group. On the other hand, the administration of different doses of extracts and 400 mg/kg of solvent fractions of the CAME decreased the increased level of those biomarkers.

#### 3.4.2. Effects of Crude Extracts and Solvent Fractions on Lipid Profiles

Administration of APAP caused a significant increase in serum levels of LDL, TC, and TGs and a significant decrease in HDL levels (Table 3). However, administration of both crude extracts at different doses and solvent fractions of CAME at 400 mg/kg resulted in the decrement of those values that have been increased by APAP.

### 3.5. Histopathological Analysis

**Figure 2:**
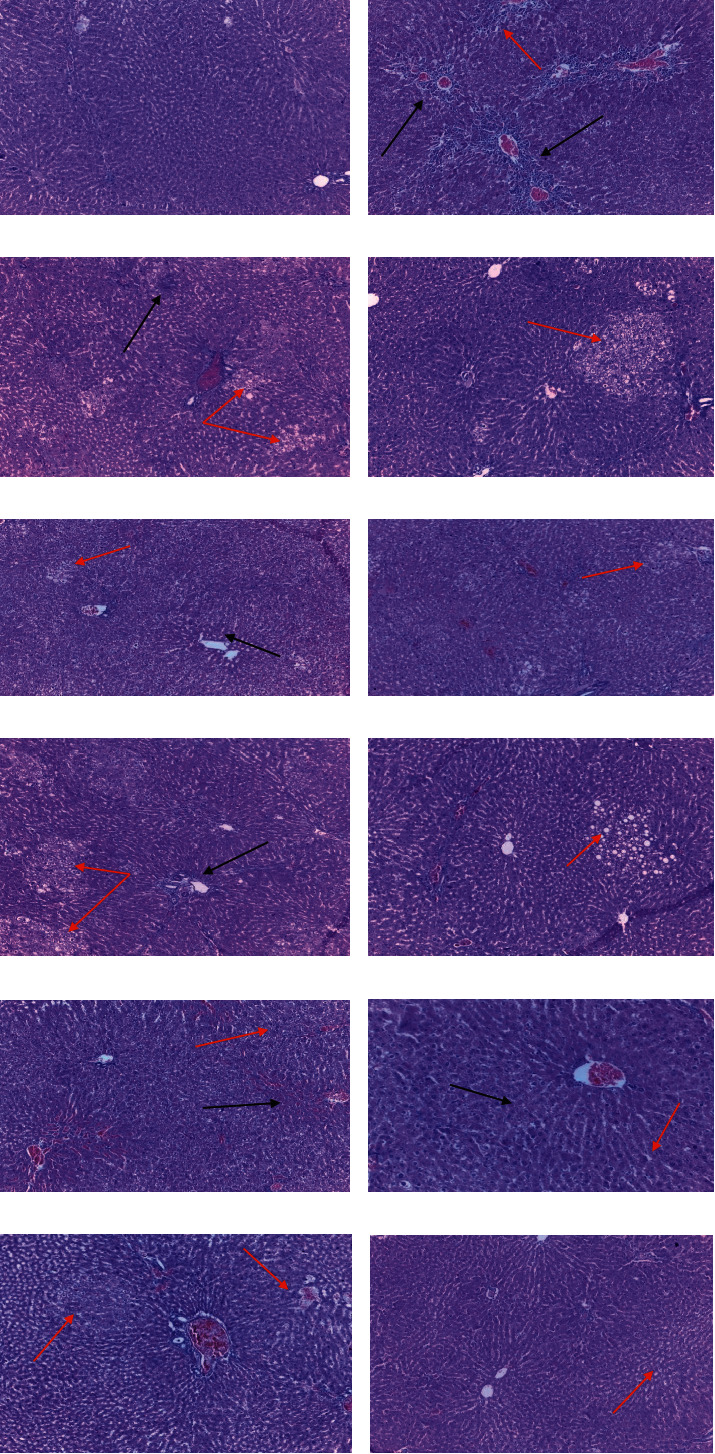
Microscopic pictures of the liver sections from different groups. *Note.* (a) Normal control group: rats treated with 2% Tween-80 followed by NS, (b) negative control group: rats received APAP alone, (c) rats received 100 mg/kg CAME, (d) rats received 200 mg/kg CAME, (e) rats received 400 mg/kg CAME, (f) rats received 100 mg/kg CAAE, (g) rats received 200 mg/kg CAAE, (h) rats received 400 mg/kg CAAE, (i) rats received 400 mg/kg CAAF, (j) rats received 400 mg/kg CABF, (k) rats received 400 mg/kg CACF, and (l) positive control group: rats received 100 mg/kg silymarin. Black arrows indicate areas of tissue necrosis and red arrows indicate areas of vacuolar degeneration of varying degree. APAP: acetaminophen, CAAE: aqueous extract of Cordia africana, CAAF: aqueous fraction of Cordia africana, CABF: butanol fraction of Cordia africana, CACF: chloroform fraction of Cordia africana, CAME: 80% methanol extract of Cordia africana, mg/kg: milligram per kilogram, and NS: normal saline.

The normal architecture of the liver was significantly altered in the negative control group that received 2 g/kg APAP compared to the normal control (Figure 2). However, preadministration of CAAE and CAME resulted in the protection of the tissue to varying extents as listed in Table 4.

### 3.6. Effects of Extracts and Solvent Fractions of Methanol Extract on SPB-Induced Sleeping Time

Administration of 150 mg/kg SPB prolonged sleep duration in the animals treated with 2 g/kg APAP from 75.70 ± 6.60 to 161.33 ± 9.70. Pretreatment of the animals with silymarin, crude extracts, and solvent fractions restored the normal sleep duration (Table 5).

## 4. Discussion

*C. africana* Lam (Boraginaceae) is a deciduous forest tree with various uses as medicine, fodder, food, and fuel [11]. The broad medicinal uses of the plant *C. africana* by different ethnic groups in Ethiopia are witnessed by the consistency in the reports of different published ethnomedicinal studies. Different parts of the plant have been used for the liver disorders in different preparations. Drinking an aqueous decoction of the dried powdered stem bark [12] and dried powdered stem bark boiled with milk [15], inhaling boiled leaves [14], and drinking water mixture of dried ground root [16] have been used for the treatment of different forms of the liver disease. Secondary metabolites such as alkaloids, flavonoids, phenols, and tannins present in both an aqueous and the methanol stem bark extracts of the plant *C. africana* have shown a promising hepatoprotective effect.

Acetaminophen (APAP) was selected to induce the liver injury to assess hepatoprotective effect in this study for two reasons. First, the model has its own prescreening lethality test, which serves as a prestudy test. Second, APAP induces hepatotoxicity, which resembles the liver injury in humans [35]. Administration of both extracts of *C. africana* stem bark showed no mortality and signs of toxicity, which might justify the edibility of the pulp of mature fruit of the plant to human beings in Ethiopia [36]. Reduction in APAP-induced lethality, which is in agreement with that of a previous study [37], could be attributed to the hepatoprotective effects of the extracts. This might be associated with inflammation and oxidative stress modulating activities of the extracts [38], that is, anti-inflammatory and antioxidant activities of the plant [22].

The decreased lethality effect of the extracts observed in the present study led to subsequent investigations carried out to confirm further hepatoprotective effects. The optimum dose of APAP (2 g/kg), which induced the liver damage, was determined to be 2 g/kg through the selection of three doses from previous studies, which was a similar procedure used in other studies [28, 39]. In the present study, administration of 2 g/kg APAP resulted in significant elevation of serum levels ALT, AST, ALP, TB, lipid profiles, LDL, TC, and TGs and decreased serum level of HDL, which was in agreement with the previous study [40]. These findings are also similar to those from other studies [41, 42]. The increase in serum levels of the liver enzymes is due to the damage of hepatocytes by the APAP metabolite, N-acetyl-p-benzoquinone imine (NABQI), which causes rupture of the cell membrane resulting in leakage of the enzymes from hepatocytes to the serum [43, 44].

Lipid metabolizing incapability of the liver caused by APAP intoxication results in abnormally high levels of LDL, TG, and TC and low levels of HDL, which would otherwise be metabolized to bile acids and transported into and stored in other body parts. After the liver injury, bilirubin cannot appropriately conjugate, resulting in higher serum levels of total bilirubin [45]. Preadministration of the standard drug, silymarin 100 mg/kg, significantly (*P* < 0.5) prevented HDL levels from getting decreased and other biomarkers from getting increased by APAP administration. Silymarin is well known for its hepatoprotective activity, which has been widely used as a standard drug in several similar studies. It revealed protective activities in many previous studies, which used APAP [40] or other liver-damaging chemicals [46]. It has been confirmed by Papackova et al. [47] that free radical scavenging properties of silymarin account for its hepatoprotective activity.

The 80% methanol extract of *C. africana* stem bark showed better activity than the aqueous extract. This might be attributed to the polarity difference of the solvents in which hepatoprotective components might have been extracted [48, 49]. CAME significantly reduced serum levels of ALT and AST dose dependently, while CAAE did the same for AST, ALP, and TGs. This might be due to the increment in the concentrations of responsible constituents with dose and/or to the strong role that the three enzymes play in biomarker activity. However, CAME has shown a dose-dependent decrease in percent protection in terms of ALP and TGs, which could be due to some toxic phytoconstituents in the extract, whose concentration increased with dose. Percent protections of 200 mg/kg CAME, 100 mg/kg CAAE, and 400 mg/kg CABF in terms of serum TB levels were found to be more than that of the standard drug, 100 mg/kg silymarin. However, the mean difference in serum TB level between the extracts and silymarin was not significant (*P* > 0.5). The same was true for serum levels of ALP and LDL, which seem to be more reduced by 400 mg/kg CACF than silymarin.

As CAME showed a better activity at medium dose, it was further fractionated with three solvents among which 400 mg/kg CACF resulted in the highest percent protection in almost all biomarkers, such as ALT, AST, ALP, HDL, LDL, TC, and TG. On the other hand, CAAF has shown better activities in percent protection of HDL, ALT, AST, and TGs than CABF. This is similar to studies by Anyasor et al. [50], in which an aqueous fraction of methanol extract of *Costus afer* leaves showed the highest hepatoprotective effect in all three different doses in diethyl nitrosamine-induced liver carcinoma in rats. However, CABF has shown the highest percent protection in terms of TB than others and better activity than CAAF in terms of ALP, LDL, and TC. All these variations could be due to the differences in composition among the fractions in which CACF contained more responsible constituents and offered it the highest hepatoprotective effects than others, which might attribute to the polarity differences among the solvents.

Histopathological examination was done to confirm the findings of biochemical analysis. In addition to the liberation of the liver enzymes, as APAP also induces oxidative stress followed by necrosis, cellular damage can also occur [51]. Additionally, many other cellular processes such as endoplasmic reticulum stress, autophagy, sterile inflammation, microcirculatory dysfunction, and liver regeneration have been identified to be involved in the pathogenesis of APAP-induced liver injury [44]. Necrosis, vacuolar degeneration, and inflammatory cell infiltration were also seen after APAP administration [52]. Degrees of both necrosis and vacuolar degeneration were higher in the negative control group, which received only APAP compared with a normal control group in the present study. However, the damages were found to be lower in the groups that received silymarin and test substances. The findings of the histopathological investigations were in agreement with those of biochemical findings, thereby confirming the hepatoprotective effect of the extracts and fractions.

SPB-induced sleeping time was used to assess the metabolizing activity of the liver. It is metabolized by hepatic microsomal drug-metabolizing enzymes (MDME), cytochrome p-450, to inactive metabolites. In the liver injury, the metabolizing activities of the liver become defected and the plasma level of the drug remains higher, which results in prolongation of sleep duration. The duration of SPB-induced sleep in the animal is considered a reliable index for the activity of hepatic MDME. The damage caused by APAP to hepatocytes causes loss of drug-metabolizing capacity of the liver, resulting in prolongation of SPB-induced sleeping time [34]. Both extracts and solvent fractions of CAME shortened SPB-induced sleeping time, which could further confirm the hepatoprotective effects of the plant. The sleeping time of the animals that received CAME and CAAE without APAP was almost similar to the control group that received SPB alone, confirming that the plant has no enzyme inhibitory activity and the sleeping time prolongation was caused merely by APAP. Moreover, CAME reduced the sleeping time compared to the standard treatment, silymarin 100 mg/kg. This is similar to the biochemical findings in which CAME showed significant and dose-dependent activities. Among the solvent fractions, CAAF showed the highest percent protection perhaps due to the variability in the concentration and activity of hepatoprotective secondary metabolites in the fractions, which accounted for the differences in hepatoprotective effects.

APAP induces the liver injury through its free radical metabolite, NABQI, which could be counteracted by the antioxidant [21] and anti-inflammatory [22] activities of the stem bark extracts of *C. africana*. Furthermore, secondary metabolites such as alkaloids, flavonoids, phenols, and tannins, which have shown hepatoprotective effects in other studies, were found also in both an aqueous [53] and methanol [22, 54] stem bark extracts of *C. africana.* Therefore, the hepatoprotective effects of *C. africana* in the present study could be associated with the anti-inflammatory and/or antioxidant activities of the constituents present in the extracts and fractions.

## 5. Conclusion

The results of the present study revealed that 80% methanol and an aqueous extract, as well as solvent fractions of the methanol extract, possess hepatoprotective activities. The present findings might provide scientific justification for the traditional claim of the use of *C. africana* for different forms of the liver disease. However, the present study did not investigate the reversal effect of the test substances on the already injured liver and it uses only a single dose of APAP to induce liver injury, which could be the future research's goals.

## Figures and Tables

**Table 1:** Effects of different doses of APAP on serum levels of the liver enzymes (ALT and AST).

Group	ALT (U/L)	AST (U/L)
NS, 10 ml/kg	58.75 ± 23.1	134.20 ± 32.03
APAP, 640 mg/kg	58.00 ± 19.4^cd^	134.65 ± 35.02^cd^
APAP, 1000 mg/kg	118.50 ± 30.9^abd^	333.50 ± 25.03^abd^
APAP, 2000 mg/kg	151.95 ± 33.9^abc^	390.05 ± 30.03^abc^

*Note.* Results are expressed as mean ± SEM; compared with ^a^10 ml/kg NS, ^b^640 mg/kg APAP, ^c^1000 mg/kg APAP, and ^d^2000 mg/kg APAP. *P* < 0.05. ALT: alanine aminotransferase, APAP: acetaminophen, AST: aspartate aminotransferase, mg/kg: milligram per kilogram, NS: normal saline, SEM: standard error of mean, and U/L: units per liter.

**Table 2:** Effects of crude extracts and solvent fractions of the 80% methanol extract of *C. africana* stem bark on serum levels of the liver enzymes and TB of rats orally administered 2 g/kg APAP (mean ± SEM).

Group	ALT (U/L)	AST (U/L)	ALP (U/L)	TB (mg/dL)
Normal control	101.57 ± 45.88	218.30 ± 73.18	118.00 ± 42.31	0.09 ± 0.02
Negative control	227.80 ± 40.80^a^	417.73 ± 67.10^a^	248.33 ± 34.55^a^	0.19 ± 0.05^a^
100 mg/kg, CAME	110.47 ± 35.18^b,d,e,f^ (92.9)	347.53 ± 70.28^b,d,e^ (35.2)	201.00 ± 44.65^b,d,e,f^ (36.3)	0.15 ± 0.01^b^ (40)
200 mg/kg, CAME	108.62 ± 40.72^b,c,e,g^ (94.4)	292.23 ± 63.38^b,c,e^ (62.9)	225.50 ± 42.31^b,c,e,g^ (17.5)	0.11 ± 0.01^b,g^ (80)
400 mg/kg, CAME	102.75 ± 39.74^b,c,d,h^ (99.1)	254.33 ± 53.20^b,c,d^ (81.9)	228.50 ± 32.30^b,c,d^ (15.2)	0.14 ± 0.01^b^ (50)
100 mg/kg, CAAE	121.96 ± 35.54^b^ (83.8)	324.77 ± 73.18^b,g,h^ (46.6)	248.00 ± 34.55^b,g,h^ (0.3)	0.11 ± 0.01^b^ (80)
200 mg/kg, CAAE	178.95 ± 56.19^b^ (38.7)	261.45 ± 80.68^b,f,h^ (78.4)	245.00 ± 40.33^b,f,h^ (2.6)	0.15 ± 0.01^b^ (40)
400 mg/kg, CAAE	153.15 ± 39.74^b^ (59.1)	254.93 ± 71.53^b,f,g^ (81.6)	219.67 ± 44.50^b,f,g^ (22)	0.13 ± 0.02^b^ (60)
400 mg/kg, CAAF	113.50 ± 56.20^b,j^ (90.5)	263.38 ± 63.38^b,j^ (77.4)	248.00 ± 37.64^b^ (0.3)	0.13 ± 0.01^b^ (60)
400 mg/kg, CABF	116.80 ± 45.88^b^ (87.9)	305.58 ± 54.30^b^ (56.2)	193.67 ± 33.13^b,i^ (41.9)	0.11 ± 0.02^b,i,k^ (80)
400 mg/kg, CACF	103.10 ± 61.44^b,i,j^ (98.8)	242.00 ± 71.27^b,i,j^ (88.1)	128.50 ± 50.10^b,i,j^ (91.9)	0.13 ± 0.02^b,j^ (60)
100 mg/kg, silymarin	98.50 ± 45.88 ^b,c,d,e,f,g,h,i,j,k^ (102.4)	241.75 ± 89.62^b,c,d,e,f,g,h,i,j,k^ (88.2)	177.67 ± 34.55^b,c,d,e,f,g,h,i,j,k^ (54.2)	0.12 ± 0.01^b,c,d,e,f,g,h,i,j,k^ (70)

Compared with ^a^normal control, ^b^negative control, ^c^CAME100, ^d^CAME200, ^e^CAME400, ^f^CAAE100, ^g^CAAE200, ^h^CAAE400, ^i^CAAF, ^j^CABF, and ^k^CACF. *P* < 0.05. ALP: alkaline phosphatase, ALT: alanine aminotransferase, AST: aspartate aminotransferase, CAAE: aqueous extract of Cordia africana, CAAF: aqueous fraction of Cordia africana, CABF: butanol fraction of Cordia africana, CACF: chloroform fraction of Cordia africana, CAME: 80% methanol extract of Cordia africana, g/kg: gram per kilogram, mg/dl: milligram per deciliter, mg/kg: milligram per kilogram, TB: total bilirubin, and U/L: units per liter.

**Table 3:** Effects of crude extracts and solvent fractions of the 80% methanol extract of *C. africana* stem bark on lipid profiles of rats orally administered 2 g/kg APAP (mean ± SEM).

Group	HDL (mg/dL)	LDL (mg/dL)	TC (mg/dL)	TGs (mg/dL)
Normal control	23.13 ± 1.65	8.40 ± 2.05	38.60 ± 4.02	61.55 ± 14.02
Negative control	19.90 ± 3.44^a^	13.03 ± 1.70^a^	45.95 ± 3.30^a^	114.35 ± 12.14^a^
100 mg/kg, CAME	23.10 ± 2.20^b,f^ (99.1)	10.20 ± 1.34^b^ (61.1)	42.85 ± 2.66^b^ (42.2)	81.78 ± 15.48^b,d,e^ (61.7)
200 mg/kg, CAME	21.24 ± 1.70^b,g^ (41.5)	11.80 ± 1.30^b^ (26.6)	42.40 ± 3.58^b,g^ (48.3)	82.40 ± 14.00^b,c,e^ (60.5)
400 mg/kg, CAME	22.92 ± 1.90^b,h^ (93.5)	10.40 ± 2.00^b^ (56.8)	42.83 ± 2.48^b^ (42.4)	87.50 ± 11.76^b,c,d^ (50.8)
100 mg/kg, CAAE	22.58 ± 1.56^b^ (83)	9.50 ± 2.05^b^ (76.2)	42.43 ± 2.05^b^ (47.9)	78.35 ± 10.86^b,g,h^ (68.2)
200 mg/kg, CAAE	21.10 ± 2.25^b^ (37.2)	11.10 ± 2.14^b^ (41.7)	42.63 ± 2.00^b^ (45.2)	75.78 ± 7.94^b,f,h^ (73.0)
400 mg/kg, CAAE	21.80 ± 2.20^b^ (58.8)	9.00 ± 1.44^b^ (87.0)	41.48 ± 2.61^b^ (60.8)	74.52 ± 9.20^b,f,g^ (75.4)
400 mg/kg, CAAF	22.80 ± 1.90^b,j^ (89.8)	12.50 ± 1.60^b^ (11.4)	45.30 ± 3.11^b^ (8.8)	77.40 ± 30.39^b,j^ (70.0)
400 mg/kg, CABF	22.28 ± 2.12^b^ (73.7)	10.10 ± 2.70^b,i^ (63.3)	43.30 ± 4.32^b,i^ (36.1)	81.45 ± 21.38^b^ (62.3)
400 mg/kg, CACF	23.03 ± 2.20^b,i,j^ (96.9)	9.38 ± 2.92^b,i,j^ (78.8)	40.05 ± 4.02^b,i,j^ (80.3)	69.50 ± 6.98^b,i,j^ (84.9)
100 mg/kg, silymarin	23.12 ± 1.85 ^b,c,d,e,f,g,h,i,j,k^ (99.70)	9.43 ± 1.51 ^b,c,d,e,f,g,h,i,j,k^ (77.8)	39.85 ± 4.02 ^b,c,d,e,f,g,h,i,j,k^ (82.9)	61.63 ± 8.11 ^b,c,d,e,f,g,h,i,j,k^ (99.8)

Compared with ^a^normal control, ^b^negative control, ^c^CAME100, ^d^CAME200, ^e^CAME400, ^f^CAAE100, ^g^CAAE200, ^h^CAAE400, ^i^CAAF, ^j^CABF, and ^k^CACF. *P* < 0.05. HDL, high-density lipoprotein; LDL, low-density lipoprotein; TC, total cholesterol; TGs, triglycerides; CAAE, aqueous extract of C. africana; CAME, 80% methanol extract of C. africana; CABF, butanol fraction of C. africana; CAAF, aqueous fraction of C. africana; CACF, chloroform fraction of C. africana, mg/kg, milligram per kilogram; mg/dl, milligram per deciliter.

**Table 4:** Effects of different doses of crude extracts and solvent fractions of the methanol stem bark extracts of *C. africana* on APAP-induced liver injury.

Group	Necrosis	Vacuolar degeneration
Normal control, NS	—	—
Negative control	+++	++
100 mg/kg, CAME	+	++
200 mg/kg, CAME	—	+
400 mg/kg, CAME	+	+
100 mg/kg, CAAE	—	+
200 mg/kg, CAAE	+	++
400 mg/kg, CAAE	—	+
400 mg/kg, CAAF	—	+
400 mg/kg, CABF	+	+
400 mg/kg, CACF	—	+
100 mg/kg, silymarin	—	—

—, normal; +, mild effect; ++, moderate effect; +++, severe effect. APAP: acetaminophen, CAAE: aqueous extract of Cordia africana , CAAF: aqueous fraction of Cordia africana, CABF: butanol fraction of Cordia africana, CACF: chloroform fraction of Cordia africana, CAME: 80% methanol extract of Cordia africana, mg/kg: milligram per kilogram, NS: normal saline.

**Table 5:** Effects of high doses (400 mg/kg) of crude extracts and solvent fractions of methanol extract on SPB-induced sleeping time in rats orally administered 2 g/kg APAP (mean ± SEM).

Group	Sleep duration (minutes)
Normal control, vehicle + SPB	75.70 ± 6.60
Negative control, APAP + SPB	161.33 ± 9.70^a^
CAME + SPB	80.25 ± 11.44
CAAE + SPB	75.80 ± 12.00
CAME + APAP + SPB	91.60 ± 4.73^b,d,e,f^ (81.4)
CAAE + APAP + SPB	129.30 ± 3.06^b^ (37.4)
CAAF + APAP + SPB	102.80 ± 5.70^b,f,g^ (68.4)
CABF + APAP + SPB	128.50 ± 7.94^b^ (38.3)
CACF + APAP + SPB	132.80 ± 5.44^b^ (33.3)
Silymarin, 100 mg/kg + APAP + SPB	85.25 ± 5.70^b,d,e,f,g^ (88.8)

Compared with ^a^normal control, ^b^negative control, ^c^CAME, ^d^CAAE, ^e^CAAF, ^f^CABF, and ^g^CACF. Numbers in the bracket show percent protection. APAP: acetaminophen, CAAE: aqueous extract of Cordia africana, CAAF: aqueous fraction of Cordia africana, CABF: butanol fraction of Cordia africana, CACF: chloroform fraction of Cordia africana, CAME: 80% methanol extract of Cordia africana, mg/kg: milligram per kilogram, NS: normal saline, and SPB: sodium pentobarbital.

## Data Availability

The data used to support the findings of this study are available from the corresponding author upon request.
